# Public health and system approach in eliminating disparities in hypertensive disorders and cardiovascular outcomes in non-Hispanic Black women across the pregnancy life course

**DOI:** 10.1016/j.ahjo.2024.100445

**Published:** 2024-08-28

**Authors:** Rachel M. Bond, Natalie A. Bello, Annette Ansong, Keith C. Ferdinand

**Affiliations:** a Women's Heart Health, Dignity Health, Arizona, 3240 S Mercy Road Suite 312, Gilbert, AZ 85287, United States of America; b Smidt Heart Institute, Cedars Sinai Medical Center, 127 S San Vincente BLVD Suite A3100, Los Angeles, CA 90048, United States of America; c Children's National Hospital, 111 Michigan Avenue, NW, Washington, DC 20010, United States of America; d John W. Deming Department of Medicine, Tulane University School of Medicine, 1430 Tulane Avenue, #8548, New Orleans, LA 70112, United States of America

**Keywords:** Maternal health, Health disparities, Social determinants of health, Cardioobstetrics, Black women, Life course

## Abstract

Hypertension is one of the leading risk factors for cardiovascular disease. The ACC/AHA/Multisociety hypertension guideline covered all aspects of the recommendations for optimal blood pressure diagnosis and management to improve cardiovascular outcomes. Despite this, there remains a growing prevalence of hypertension within the United States, largely in non-Hispanic Black women at earlier stages of their life course. This highlights the evident racial disparities, but offers a targeted opportunity for improved outcomes. With hypertension increasingly seen in the antenatal and immediate postpartum period, and obstetrics societies weighing in on the need to alter pharmacotherapy initiation goals, national initiatives have purposefully targeted pregnant and postpartum women in an effort to improve outcomes. This same energy must also re-focus health care efforts across the entire health continuum. Public health and system strategies are in place to do so, with the strongest enforcing initiatives as early as childhood with a greater focus on primordial prevention.

## 1 Introduction

**Fig. 1:**
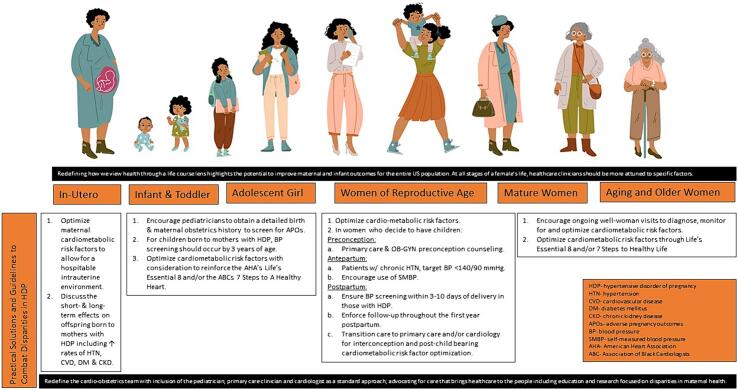
Eliminating disparities in hypertensive disorders and cardiovascular outcomes in Black women across the pregnancy life course.

In the United States (US), hypertension (HTN) prevalence is higher in non-Hispanic Black (NHB) women than Hispanic or non-Hispanic White (NHW) women [1]. Similarly, in women of childbearing age, rates of hypertensive disorders of pregnancy (HDP) such as chronic (HTN that precedes pregnancy or occurs prior to 20 weeks gestation) and gestational HTN (de novo hypertension after 20 weeks of gestation) and/or preeclampsia are highest among NHB women, and are increasing [[2], [3], [4]]. Hypertension is the most commonly encountered comorbidity by obstetricians, and is a leading cause of severe maternal morbidity (SMM) [5], and mortality [[6], [7], [8]]. Rates of SMM and mortality [5] have been steadily increasing in the US over the past several decades. This trend is in direct opposition to both global trends in maternal health resulting in a decline in maternal mortality by more than a third from 2000 to 2017, and dramatic advances in perinatal care reducing infant morbidity and mortality worldwide (Fig. 1).

In light of these disparities in the US, national initiatives including the Million Hearts 2027 have targeted pregnant and postpartum women with HTN [9]. Such efforts are in place to prevent the short- and long-term adverse pregnancy outcomes (APOs). Nevertheless, current endeavors have proven to be suboptimal and SMM and mortality rates continue to climb. This review will discuss potential solutions from public health and system approaches that may be more beneficial by focusing on primordial care and viewing health through the scope of the life course.

## 2 Preconception: disparities in offspring born to mothers with HDP

### 2.1 In-utero to adolescence

The primary focus of HDP has been on the mother and her future risk for cardiovascular disease (CVD) [10,11]. Women with a history of HDP are two times as likely to suffer from CVD 10–15 years after childbirth [12,13]. This same concern and advocacy should be extended to the fetus, infant and child born to mothers with HDP [2,14].

It is well studied that HTN and its related outcomes disproportionately affect NHB adults [15,16]. The same can be said for NHB children, with a 29 % increased odds of HTN compared to their NHW counterparts. Comparably, NHB infants have a higher prevalence of low birthweight (LBW), and obesity [17]. With LBW the leading cause of infant mortality, the infant mortality rate is 2.3 times higher in NHB than NHW infants; and hence NHB infants are 4 times more likely to die from LBW and its complications than NHW infants [14,[18], [19], [20]].

The direct manifestations of preeclampsia on the fetus and newborn include preterm delivery, intrauterine growth restriction (IUGR), placental abruption, and stillbirth. The morbidities for an infant born prematurely or IUGR can be dire and may result in a prolonged hospitalization in the neonatal intensive care unit [21,22].

On a granular level, preeclampsia has been noted to cause an increase in oxidative stress biomarkers, possibly impacting endothelial cell dysfunction [21,23]. The oxidative stress seen may be carried into the fetus. In addition to an increase in free radicals and decrease in antioxidants, there may be increased cholesterol, triglycerides, low density lipoprotein and lowered high density lipoprotein perhaps suggestive of the proatherogenic state initiated in neonates and a potential reason for their risk of developing CVD [21,24].

Several studies have shown that the offspring of mothers with HDP have a tendency to become hypertensive adults [25,26]. The ‘Barker hypothesis’ demonstrates that LBW infants have higher rates of CVD, diabetes and chronic kidney disease in adulthood. This may occur from adjustments that the fetus undergoes due to undernourishment from placental insufficiency [22,[27], [28], [29]].

A population-based study of Nordic infants followed from 1973 to 2014 looked over 5.8 million singleton live births, where 218,322 infants (3.76 %) were born to mothers with HDP. Of these, there was an increased likelihood of stroke (33 %) and heart disease (29 %) with a risk that lasted up to 41 years of age [30]. This highlights the vigilance required in including the obstetrical history of the mothers as part of the routine history of pediatric patients to help identify those children who may be at risk for future cardiac events. It appears increasingly evident that HDP is not a disease that simply appears in pregnancy but is connected to risk factors, many of which are modifiable before and after pregnancy that must be addressed [31].

### 2.2 Women of reproductive age

Though the factors causing preeclampsia are not fully elucidated, being aware of and optimizing as many of the identified contributing factors is key to a hospitable intrauterine environment for the fetus. Such risk factors include a prior maternal history of HDP, conception utilizing artificial reproductive technology, multifetal gestation, or diabetes. Use of aspirin for women at high risk of developing preeclampsia is advised starting between 12 and 28 weeks gestation through delivery [32]. Practitioners should especially be on high alert when treating NHB women as they are at greater risk of developing preeclampsia and experiencing maternal and infant mortality. The totality of the Black maternal health crisis must in fact include its effects on the fetus, newborn, and child.

## 3 Disparities in HDP

There are persistent racial disparities in maternal outcomes experienced by NHB women [6,7,[33], [34], [35], [36], [37], [38], [39], [40]]. In 2020, NHB women encountered 55.3 maternal deaths per 100,000 live births compared to 18.2 deaths per 100,000 live births to Hispanic and 19.1 deaths per 100,000 live births to NHW women. When examined by age category, NHB women 40 years of age and over experienced 263.1 deaths per 100,000 live births compared to 86.0 and 96.8 per 100,000 live births for Hispanic and NHW women. In women <25 years of age, those rates were 28.8, 11.5, and 7.7 per 100,000 live births for NHB, NHW, and Hispanic women respectively [3].

Cardiovascular diseases are the leading cause of maternal mortality in the US, and many of these deaths are avoidable. Cardiomyopathy, pulmonary embolism, and HDP disproportionately contribute to pregnancy-related deaths in NHB compared to NHW women, respectively accounting for 19.1 %, 11.0 %, and 22.1 % of the Black/White disparity [41]. The heightened rate of SMM and mortality experienced by NHB women persists irrespective of level of education or socioeconomic status (SES) which may in part be due to social determinants of health (SDOH), systemic racism and the cumulative burden of chronic stress and life events in addition to a higher burden of chronic medical conditions like HTN [42], which have historically suboptimal control.

Utilizing the criteria of treatment to <140/90 mmHg, blood pressure (BP) control was achieved in 66.3 % of NHW, 44.7 % of NHB, and 49.9 % Hispanic women [1]. Of the Centers for Disease Control's (CDC) 21 indicators of SMM [43], 10 are potential sequelae of HTN including peripartum cardiomyopathy (PPCM) which disproportionately impact NHB women [6,44]. Cardiomyopathy accounts for >10 % of maternal deaths and is the leading cause of postpartum death [45]. PPCM, is more common in women of African descent with NHB women experiencing more severe left ventricular systolic dysfunction compared to NHW women [46]. In US women with PPCM, there is a more than four-fold higher prevalence of preeclampsia [44]. While not specifically examined in PPCM, race-based disparities in heart failure risk factors, incidence, and prognosis have been extensively documented and likely contribute to the disproportionately higher rates of maternal mortality experienced by NHB women [47].

## 4 Postpartum disparities related to HTN

The postpartum period represents a critical window to initiate targeted interventions to improve cardiometabolic health following pregnancies complicated by HDP. Despite evidence-informed guidelines, population data demonstrate that postpartum screening rates and interventions of women who manifest such conditions remain suboptimal and vary substantially, with marked racial/ethnic disparities.

### 4.1 Postpartum hypertension

In the US, HDP account for approximately 7 % of maternal mortality, with approximately 70 % of these deaths occurring postpartum [45]. Unfortunately, data continue to demonstrate only 13.7 % of women diagnosed with a HDP, disorders including eclampsia, preeclampsia, which may be superimposed on chronic hypertension, and gestational hypertension, attend BP screening within 10 days of delivery, despite recommendations to do so [48]. BP typically declines immediately after delivery and then rises, peaking between 3 and 7 days postpartum [49]. Having close outpatient follow-up is essential to identify and treat HTN and prevent adverse effects. Data from the Pregnancy Risk Assessment Monitoring System (PRAMS), a surveillance system of the CDC and state health departments, suggest wide racial/ethnic disparities exist in the percent of women who attend a postpartum care visit, with one of the greatest disparities noted in NHB women [50]. With the incidence of de novo postpartum hypertension or preeclampsia, HDP occurring up to 42-days postpartum, ranging from 0.3 % to 27.5 % [51], ensuring access to appropriate postpartum follow-up is necessary. Delayed recognition, and subsequent lack of early treatment is associated with an increased risk of readmissions in our healthcare system due to SMM, particularly from eclampsia [52].

With postpartum HTN the leading cause of repeat hospitalization after childbirth, identifying risk factors for readmission is important. These include advanced age, SES, chronic comorbidities, multiple gestation, cesarean delivery, and other high-risk pregnancy conditions [[53], [54], [55]]. Maternal race has proven to be a major risk factor for APOs, with associated disparities in overall risk for severe morbidity and mortality [56]. More recently, a cross-sectional analysis demonstrated that NHB women were more likely to be readmitted postpartum, to suffer SMM during readmission including greater rates of preeclampsia with severe features (43.3 % vs 10.0 % non-Black, *p* = 0.01), and to suffer life threatening complications including pulmonary edema/acute heart failure [7]. NHB women with cardiometabolic risk factors may benefit from targeted short-term postpartum follow-up.

### 4.2 Post-child bearing hypertensive disorders

Oftentimes, pregnancy provides a window into an individual's future cardiovascular (CV) health. It is well-established that women with HDP have increased risk of subsequent APOs and a four times greater risk of chronic HTN and two times greater risk of CVD [57]. With untreated HTN leading to a range of disabling and potentially fatal chronic illnesses, the postpartum period is a critical time when CV lifestyle changes should be reinforced and cardiometabolic risk factors optimized for interconception or post-child bearing health [58]. The American College of Obstetricians and Gynecologists (ACOG) has released guidelines recommending that postpartum care is an ongoing process, with services and support tailored to each woman's individual needs [59]. With nearly 58 % of NHB women in the US having HTN compared to about 41 % of NHW and Hispanic women [60], targeted efforts may be appropriate to reinforce this ongoing care.

## 5 Practical solutions & guidelines to combat disparities in hypertensive disorders of pregnancy

Despite multi-society statements and guidance, there are no clear universal guidelines on how best to manage HDP as a means to combat disparities. An important consideration in optimizing maternal care is characterizing one's risk at every stage of the health continuum and improving access to, and quality of care in the most marginalized risk groups.

## 6 Lifespan approach

Numerous studies have demonstrated that primordial prevention can positively influence one's lifetime health [61]. As such, re-defining how we view health through a life course lens highlights the potential to improve maternal and infant outcomes for the entire US population [62].

Well-known factors contributing to poor and disparate maternal health outcomes in the US include SDOH centered on psychosocial stressors such as multigenerational structural racism [37]. These conditions increase the risk of common cardiometabolic risk factors such as obesity, chronic HTN and diabetes which negatively impact maternal and CV health outcomes [63]. NHB women have one of the highest prevalence of these risk factors [60], with many beginning at early stages of their life course. As such, focusing efforts to optimize care beginning as early as childhood can be one novel approach to combat APOs. Pediatricians and pediatric subspecialists can do so by screening for these risk factors as early as indicated. Pediatric providers must be attuned to recognizing aberrancies in history, physical exams and lab work that may bring a CV risk factor to the forefront. Providers must highlight the importance of children making it to their routine well-child check visits where BP should be checked, typically beginning at the age of 3 years. As mentioned earlier, the role of the maternal and infant birthing history is key and should be standardized into the history and physical exam. Furthermore, in those with a history of prematurity or LBW, it may be reasonable to consider the BP checked sooner than 3 years of age [64,65]. Once risk factors are identified, counseling on the role a heart-healthy lifestyle plays is pivotal for improved outcomes. Leading CV organizations, such as the Association of Black Cardiologists (ABC), American College of Cardiology (ACC) and the American Heart Association (AHA) have promoted prevention strategies to decrease the incidence of heart disease [66,67]. Utilizing these tools to focus on primordial prevention, can leave a lasting impact on the health of the child and the health of their potential future offspring.

### 6.1 Re-challenging the house of medicine by expanding the meaning of cardio-obstetrics

The establishment of the cardio-obstetrics team that provides coordinated healthcare has been effective in lowering CV morbidity and mortality and improving outcomes in women with congenital or acquired heart disease, high risk cardiometabolic factors and/or APOs [68]. Moving beyond its standard concept, and including the integration of primary medical and gynecological care both preconception and postpartum remains essential [69]. Beyond that, the inclusion of the pediatrician and/or pediatric subspecialist can further improve outcomes by focusing on primordial prevention [70].

Several care models exist that incorporate preventive measures to reduce cardiometabolic risk, including weight monitoring, nutritional referral, exercise recommendations, and self-monitored blood pressure (SMBP) screening [71]. As suggested by the 2017 ACC/AHA/Multisociety HTN guideline, SMBP with ambulatory or home BP cuffs is very helpful and practical to obtain out-of-office BP readings [72]. It is for this reason, the use of it should replace auscultatory methods of BP measurement as it provides a more reliable estimate of control at different times across a 24-hour period and allows for timely decisions to be made on management. To this point, its use in the antepartum period has been increasingly promoted [71], including initiatives such as Million Hearts 2027 [9]. To allow for this, Million Hearts 2027, like many efforts, promotes the expansion of Medicaid for SMBP devices.

### 6.2 Advocacy

To build on Million Hearts 2027 vision, the role of the clinician as an advocate for policy change is pivotal. This includes extending Medicaid coverage to one year postpartum; improving coverage of medications; reinforcing the need for social changes, including addressing food insecurity, access to child care and/or ability to attend both obstetric and pediatric appointments.

Similar advocacy led to the Dept. of Health and Human Services aiming to improve BP control rates above 80 % [1]. With such advocacy, a cautionary tale still remains. The state of California has been a national leader in maternal quality improvement since 2006 when the California Maternal Quality Care Collaborative formed. While SMM and mortality has declined in California, the disparity ratio not only persists but has actually increased from 3.4 to 6.0 from 2008 to 2016 the last year data was publicly available [73]. To date, the drivers of this ratio have not been identified, but it may be that a focus on hospital level interventions targeting clinicians and administrators fails to account for and address the complex socioeconomic and environmental factors underlying health disparities, thus widening the gap. This paradox underlies the need for rigorous evaluation of novel interventions that can also bring healthcare to the people via community-based solutions and ensure their outcomes are achieving the desired impact on health equity.

### 6.3 Optimize what we know: treatment timing controversies that support tighter BP control

Diagnostic BP treatment thresholds for the general population have evolved. In 2017, the ACC/AHA/Multisociety hypertension guideline lowered the threshold for the diagnosis of stage 1 hypertension to 130/80 mmHg [72]. The decision was based on observational studies and clinical trials demonstrating reduced CV events with treatment to lower levels.

Such a change regarding the threshold for initiation of antihypertensive treatment has been considered controversial for pregnant women due to a lack of certainty of its benefits for this population vs. the potential neonatal risk. Most guidelines agree HTN in pregnancy is defined as BP of ≥140/90 mmHg [74]. Such guidelines, based largely on expert opinions, including ACOG recommend treatment thresholds starting at severe values of ≥160/110 mmHg in the antepartum period and ≥150/100 mmHg in the postpartum period [75].

Although the best treatment thresholds still have yet to be determined for postpartum hypertension, retrospective cohort data remains promising. With that, the use of pharmacotherapy initiation threshold for stage 2 hypertension (140/90 mmHg) in the postpartum period, has shown to significantly improve maternal outcomes and decrease readmissions with no deleterious effect on neonatal outcomes [76]. Researchers most recently evaluated if the same positive effects can be achieved in the antenatal period if using targets for nonpregnant, reproductive-age adults. The Chronic Hypertension and Pregnancy (CHAP) study, included >2400 pregnant participants' randomly assigned labetalol or nifedipine with a treatment goal of <140/90 mmHg or no treatment unless BP is severe in patients with mild chronic HTN [77]. Results demonstrated that in pregnant women with mild chronic HTN, targeting BP of <140/90 mmHg was associated with better pregnancy outcomes, with no increase in the risk of poor neonatal outcomes.

Using the ACC/AHA/Multisociety 2017 guideline for stage 1 has improved the appropriate identification of women who may go on to develop severe HDP and have associated increased risk for adverse fetal/neonatal outcomes [78,79]. As NHB women have a higher incidence of HDP, research is needed to see if these populations would benefit the greatest from modifying the guidelines to suggest more aggressive BP monitoring and treatment thresholds.

### 6.4 The future direction of removing HDP disparities in care: where do we go from here?

We still do not fully understand the mechanistic cause for preeclampsia. We best understand that it's associated with placental dysfunction, anti-angiogenesis, oxidative stress and inflammation [80], and that there are women at higher risk for developing preeclampsia, such as NHB women. To recognize and best manage, future studies focusing on the epigenetic, transcriptomic aspects of understanding the phenotypes are essential. In the interim, educating the community along with the healthcare professionals on the risks for short- and long-term CVD is necessary and should become the standard of care. As innovative research continues in the cardio-obstetrics arena, there are steps clinicians can take to better identify women at higher risk for HDP during preconception and prenatal care visits. These same steps should be extended well past the postpartum visits.

Such steps may include broad implementation of evidence-based remote monitoring programs, which have the potential to significantly reduce adverse outcomes. A study be Hersbherg, et al [81] nicely illustrates this potential. With such programs comes a much-needed handoff for mothers at greatest risk, particularly women of color, by enabling continuous monitoring and timely intervention. Such interventions may also address in office follow-through, which may be challenging for some patients. However, more research is needed to fully understand the long-terms benefits and optimize these programs' implementation.

To date, the American Academy of Pediatrics (AAP) recommends routine screening for postpartum depression (PPD) in mothers during well-child visits at 1, 2, 4, and 6 months of age [82]. The AAP emphasized the importance of early identification and intervention for mothers with PPD in mitigating the impact on parent-child interaction and social-emotional development, making this screening the standard of care. With the frequency of check-ups coupled with the emerging doctor-family relationship, this recommendation seems rational to be included in well-baby care. Perhaps extending a similar approach by utilizing the pediatrician office to also screen for postpartum HTN is appropriate. Although this also seems logical, this is an area that will require more research and advocacy support.

## 7 Conclusion

The maternal health crisis of unacceptably high maternal morbidity and mortality is being increasingly recognized, especially racial disparities. Non-Hispanic Black women are dying at disproportionately high rates from largely preventable causes of maternal mortality, including HDP and premature CVD. There must be public health and system approaches in place to eliminate disparities in hypertensive disorders and CV outcomes in NHB women across their life course. Re-focusing health care efforts across the health continuum, starting as early as childhood with a greater focus on primordial and primary prevention is essential.

## CRediT authorship contribution statement

**Rachel M. Bond:** Writing – review & editing, Writing – original draft, Supervision, Conceptualization. **Natalie A. Bello:** Writing – original draft, Data curation, Conceptualization. **Annette Ansong:** Writing – original draft, Data curation. **Keith C. Ferdinand:** Writing – review & editing, Conceptualization.

## Declaration of competing interest

The authors declare that they have no known competing financial interests or personal relationships that could have appeared to influence the work reported in this paper.
